# Selective Magnetic Field Generation Method for Effective Manipulation of Two-Dimensional Magnetic Microrobots Using a Triad of Electromagnetic Coils

**DOI:** 10.3390/mi17030337

**Published:** 2026-03-10

**Authors:** Dongjun Lee, Yonghun Lee, Seungmun Jeon

**Affiliations:** 1 MESL, Department of Mechanical and Automotive Engineering, Kongju National University, Cheonan 31080, Republic of Korea; 2 GITECH, Kongju National University, Cheonan 31080, Republic of Korea

**Keywords:** electromagnetic actuation, magnetic microrobot, magnetic navigation system, triad of electromagnetic coils

## Abstract

This study proposes a new method for effectively manipulating a magnetic microrobot in a two-dimensional manner using a triad of electromagnetic coils (TEC). A TEC is a system consisting of three circular coils of the same type arranged in the form of a triangle. It has a simple structure and exhibits magnetic symmetry. This study sought to develop a method to more accurately manipulate and reduce the energy consumption of microrobots using a TEC. This was accomplished by selectively using individual coils of a TEC with respect to the robot’s position, moving direction, and other manipulating conditions based on the structural characteristics and magnetic field distribution pattern of the TEC. Effective calculation methods and operating procedures are also proposed. The proposed method was found to effectively generate the necessary actuation force to control microrobots by using either one or two of the coils of a TEC, depending on the given conditions. This type of process results in improved precision in magnetic field generation and a reduction in energy consumption while making it easier to control microrobots. Magnetic fields and actuation forces were generated using the proposed method under various experimental conditions, and these results were verified through simulations to confirm the validity of the proposed method. In addition, a TEC and a closed-loop control system were built and used to test the actuation of microrobots over various paths, and the results confirmed the superiority of the proposed method compared to existing methods.

## 1. Introduction

Microrobots designed with a magnetic body can be remotely operated using an external magnetic field as a power source. They can be compact in size, from a few millimeters down to several micrometers in all dimensions, making them particularly applicable as one of the least invasive approaches in various fields [1,2]. A great deal of research on microrobots has been conducted to customize their functions to suit their end purposes. Examples include in vivo microrobots for use in the human body, to perform various tasks in the vascular system [3,4,5,6,7,8], digestive system [9,10,11,12,13], and central nervous system [14,15], such as blood clot removal, stent delivery, biopsy, and drug delivery. In vitro microrobots have also been developed for various two-dimensional (2D) functions such as cell manipulation [16,17], material composition [18,19], and fluid control [20,21,22]. Additional studies have also been conducted on microrobot mechanisms when operated by various forms of magnetic fields, such as a uniform field [23,24], gradient field [25], rotating field [26], and oscillating field [27,28]. Magnetic navigation systems (MNSs) capable of efficiently generating and controlling such magnetic fields [29,30,31,32] have also been investigated.

A MNS is a system consisting of multiple coils with specific shapes, such as circular coils, square coils, and saddle coils. The coils are arranged in various geometric patterns, such as 2D polygons or three-dimensional polyhedra, to generate proper levels of actuation force suited to the structure and purpose of the particular microrobots [33,34,35,36,37,38]. Complex magnetic fields needed to manipulate the microrobots can be generated, eliminated, and adjusted by precisely controlling the current input for each of the coils that compose the MNS, and by causing the magnetic fields of each coil to overlap one another. Typically, the coils used to manipulate microrobots are designed with a relatively large size to allow a sufficient region of interest (ROI), within which a microrobot can be manipulated as desired. This design, however, tends to reduce the intensity of the applied magnetic field, and to compensate for this loss, the number of turns of coils is increased.

Various forms of MNS have been used to study manipulation methods of microrobots according to their applied use [39,40,41,42,43]. Nelson et al. revealed that a microrobot could be manipulated on a 2D plane using a single Helmholtz coil (two circular coils) and a single Maxwell coil (two circular coils) connected to an electric motor [39]. However, with this system, the coils are physically moved during operation, and this is considered to be very inefficient for manipulating microrobots intended for use in relatively large organisms, such as the human body. Choi et al. confirmed that it was possible to manipulate microrobots in a 2D manner using a MNS without physical movement of its coils, by properly arranging two pairs of Helmholtz and Maxwell coils (a total of eight coils) such that they intersected at right angles with each other [40]. However, the arrangement of these Helmholtz and Maxwell coils was designed to fulfill the required geometric conditions for their circular coils, and this combination of multiple coils required using coils with different diameters. Jeon et al. combined existing Helmholtz and Maxwell coils with newly developed gradient and uniform saddle coils into a cylindrical shape, and as a result, a MNS with a compact cylindrical structure design was obtained [41]. With this method as well, however, a total of eight coils were still required to manipulate the microrobots.

As a greater number of coils is used, it becomes more likely that the MNS will have an excessively large size for the ROI of a microrobot. This leads to reduced efficiency in magnetic field generation, thereby increasing energy consumption. Furthermore, a MNS consisting of a group of multiple coils may suffer from magnetic field generation error, which can occur due to manufacturing, arrangement, or synchronization processes of the coils. Such errors can be increased when the MNS consists of a relatively large number of coils of different sizes and shapes, which can potentially worsen the offset, asymmetry, distortion, and noise effect of the magnetic field.

**Figure 1:**
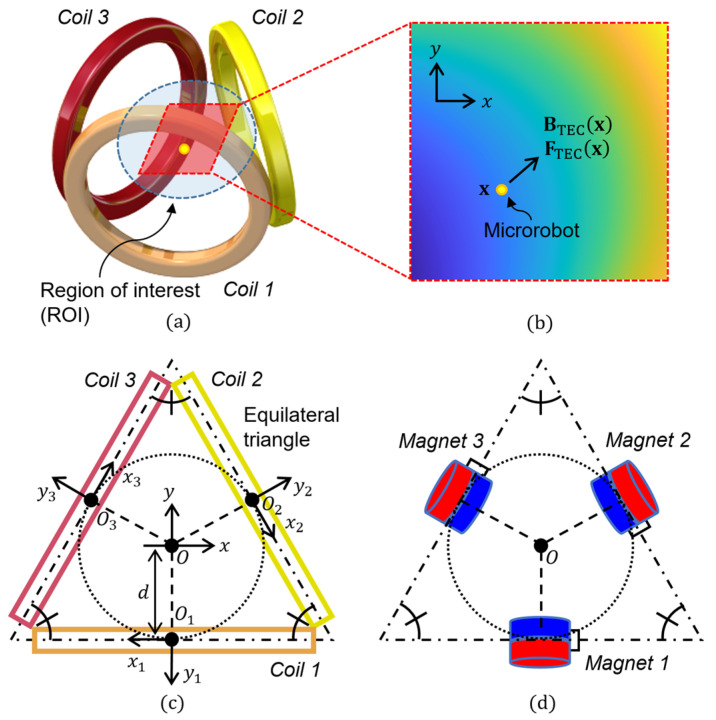
Schematic views of (**a**) TEC composed of three identical circular coils, (**b**) a microrobot located in the *xy*-plane of the TEC, (**c**) geometrical configuration of the TEC including the global and local coordinates, (**d**) a virtual magnet system analogous to the TEC shown in Figure 1c.

Go et al. demonstrated that it was possible to manipulate a microrobot in a 2D manner using a fixed MNS composed of Helmholtz and Maxwell coils (four circular coils) that intersected at right angles with one another [42]. Hwang et al. reported that the position of a microrobot could be controlled with a trapping-point control method using a single circular coil [43]. This approach employs only a single coil but requires the coil to physically move in various modes, including translation and tilting. Thus, a separate multiple-degree-of-freedom robot system is required to ensure that such movements are precisely controlled. Lee et al. showed that a microrobot could be manipulated in a 2D manner using a triad of electromagnetic coils (TEC), as shown in Figure 1a [44,45]. In a TEC system, three circular coils of the same type are symmetrically arranged and fixed in the form of a triangle. This gives it a compact structure, and its magnetic field is also symmetric. However, conventional methods for manipulating microrobots using a TEC are based on the assumption that the three coils of the TEC are used in parallel at all times. Since the input currents of the TEC are numerically calculated considering the complex equations for the magnetic force and torque generation, this assumption may result in suboptimal electrical energy efficiency, diminished manipulation accuracy, and increased control effort.

This study presents a new magnetic field generation method to further improve the efficiency and precision of microrobot manipulation using the TEC. Results demonstrated that 2D manipulation of a microrobot can be achieved by selectively using only part of the three coils of the TEC. Several conditions and calculation procedures were established to determine which coil combination and how much coil current should be applied to the TEC. In addition, the distribution of magnetic fields and actuation forces corresponding to different manipulation conditions of the microrobot was calculated and analyzed to verify the validity of the proposed method. Finally, to confirm the superiority of the proposed method, closed-loop control tests were carried out where the microrobot was moved along different paths by actuating different coil combinations of the TEC.

## 2. Generating the Actuation Force for a Magnetic Microrobot

### 2.1. Magnetic Field and Magnetic Force

The magnetic torque and magnetic force exerted on a microrobot located in a magnetic field can be expressed, respectively, as follows:

(1) T=m×B,

(2) $$F=\nabla \left(m\cdot B\right)=\frac{\partial B}{\partial X}m.$$

Here, m, B, $\frac{\partial B}{\partial X}$, T, and F refer to the magnetic moment of the microrobot, the magnetic field, the Jacobian matrix of the magnetic field with respect to a Cartesian coordinates system (X), the magnetic torque, and the magnetic force, respectively. The magnetic field generated by a circular coil can be analytically calculated using Biot-Savart’s law [46]. Since a TEC has a symmetric structure, the magnetic field generated by a TEC at a certain position x with respect to the geometric center (O) of the TEC as the reference, as well as its Jacobian matrix, can be expressed as the following equations using the local coordinate system and transformation matrix of each coil of the TEC.

(3) $$B_{\mathrm{TEC}}=\sum_{k=1}^{3}R_{z}^{\theta_{k}}B_{k}^{O_{k}}\left(x_{k}\right)=A_{B}\left(x\right)I_{\mathrm{TEC}}$$

(4) $$\frac{\partial B_{\mathrm{TEC}}}{\partial X}=\sum_{k=1}^{3}R_{z}^{\theta_{k}}\frac{\partial B_{k}^{O_{k}}\left(x_{k}\right)}{\partial X_{k}}R_{z}^{{-\theta }_{k}}=J_{B}\left(x,I_{\mathrm{TEC}}\right)$$

Here, $B_{k}^{O_{k}}$, $\theta_{k}$, $R_{z}^{\theta_{k}}$, $R_{z}^{{-\theta }_{k}}$, $x_{k}$, $X_{k}$, x, X, $A_{B}$, $J_{B}$, and $I_{\mathrm{TEC}}$ refer to the magnetic field generated by the *k*th coil of the TEC with respect to the local coordinate system ($O_{k}$) as the reference, the rotation angle of the *k*th coil with respect to the global coordinate system (O), the rotational transformation matrix in the *z*-axis direction ($\theta_{k}$, ${-\theta }_{k}$), the relative position of the microrobot with respect to the local coordinate system of the *k*th coil, the local coordinate system of the *k*th coil, the position of the microrobot with respect to the global coordinate system, the global coordinate system, the actuation matrix with respect to the magnetic field of the TEC and its Jacobian matrix, and the current vector exerted on the TEC, respectively. The positional relationship between each circular coil and the microrobot can be expressed as follows:

(5) $$\theta_{k}=\frac{\pi \left(2k+1\right)}{3},$$

(6) $$x_{k}=R_{z}^{{-\theta }_{k}}\left(x+dR_{z}^{\theta_{k}}\hat{j}\right).$$

Here, d and $\hat{j}$ refer to the distance between the center point of the TEC and the center of the circular coil and the vertical-direction unit vector at the center of each circular coil, respectively. In general, when an external magnetic field is exerted on a microrobot, the microrobot is then subjected to a magnetic torque that induces the magnetic moment of the microrobot to align with the applied external magnetic field. Therefore, provided the microrobot can rotate as desired within a plane, as shown in Figure 1, it can be assumed that the magnetic moment of the microrobot will always be parallel to the direction of the applied external magnetic field. Based on this relationship, the magnetic force generated by the TEC can be expressed using (2) and (4) as a function of the current of the TEC:

(7) $$F_{\mathrm{TEC}}=\frac{\partial B_{\mathrm{TEC}}}{\partial X}m=J_{B}\left(x,I_{\mathrm{TEC}}\right)\left[\begin{array}{c} m_{0}cos\alpha_{B}\\ m_{0}sin\alpha_{B}\\ 0 \end{array}\right]=A_{F}\left(x\right)I_{\mathrm{TEC}}.$$

Here, $m_{0}$, $\alpha_{B}$, and $A_{F}$ refer to the magnitude of m, the direction of the external magnetic field, and the actuation matrix with respect to the magnetic force, respectively. Thus, the 2D magnetic field and magnetic force generated by the TEC on the *xy*-plane can be expressed as follows:

(8) $$\left[\begin{array}{c} B_{\mathrm{TEC}}^{xy}\\ F_{\mathrm{TEC}}^{xy} \end{array}\right]=\left[\begin{array}{c} B_{0}cos\alpha_{B}\\ B_{0}sin\alpha_{B}\\ F_{0}cos\alpha_{F}\\ F_{0}sin\alpha_{F} \end{array}\right]=\left[\begin{array}{c} A_{B}^{xy}\left(x\right)\\ A_{F}^{xy}\left(x\right) \end{array}\right]I_{TEC}.$$

Here, $A_{B}^{xy}$, $A_{F}^{xy}$, $B_{0}$, $F_{0}$, and $\alpha_{F}$ are the actuation matrix with respect to the magnetic field and magnetic force on the *xy*-plane, the magnitude of the magnetic field and magnetic force, and the direction of the magnetic force, respectively. The 2D movement of the microrobot can be controlled by adjusting the current exerted by the TEC on each coil according to (8). Nonetheless, (8) is an overdetermined equation, and thus the $I_{TEC}$ that fulfills the given x, $B_{0}$, $\alpha_{B}$, $F_{0}$, and $\alpha_{F}$ conditions may not exist.

However, based on the previously discussed assumption that the alignment direction of the microrobot is always parallel to the direction of the applied magnetic field, it can be reasoned that the direction and magnitude of the magnetic field are dependent on those of the microrobot’s magnetic force. Therefore, (8) can be reduced to a simpler form, as a function of the magnetic force, as follows:

(9) $$F_{\mathrm{TEC}}^{\mathrm{xy}}=\left[\begin{array}{c} F_{0}\cos\alpha_{F}\\ F_{0}\sin\alpha_{F} \end{array}\right]=\left[\begin{array}{ccc} A_{F}^{x_{1}}\left(x\right) & A_{F}^{x_{2}}\left(x\right) & A_{F}^{x_{3}}\left(x\right)\\ A_{F}^{y_{1}}\left(x\right) & A_{F}^{y_{2}}\left(x\right) & A_{F}^{y_{3}}\left(x\right) \end{array}\right]\left[\begin{array}{c} i_{1}\\ i_{2}\\ i_{3} \end{array}\right]=A_{F}^{\mathrm{xy}}\left(x\right)I_{TEC}.$$

Here, $i_{k}$ is the current of the *k*th coil of the TEC. (9) describes a situation in which the 2D movement of the microrobot is controlled by adjusting the magnitude and direction of the magnetic force only. Given that the value of the actuation matrix in (9) varies depending on the position of the microrobot, the solutions of (9) are nonlinearly distributed. Therefore, (9) can be solved by numerically determining a local minimum considering the allowable range of current that can be used as an input for the TEC. In the present study, parts of the three coils of the TEC were selectively used to effectively manipulate a microrobot. Accordingly, the actuation matrix in (9) can be further reduced to a simpler form that only considers cases where any component of the current vector is not zero.

### 2.2. Proposed Method

In general, the magnetic field generated by a circular coil has a distribution similar to that of a permanent magnet [46]. In this light, a TEC can be regarded as being composed of three fixed virtual magnets whose magnetic moment strengths can be independently adjusted (Figure 1d). As a result, it can be assumed that an actuation force is induced in the microrobot by a combination of the attractions exerted by the three virtual magnets. Based on this mechanism, one may assume that 2D actuation of a microrobot can be accomplished by selectively using a part of the coils (virtual magnets) of the TEC under each of the following conditions.

**Figure 2:**
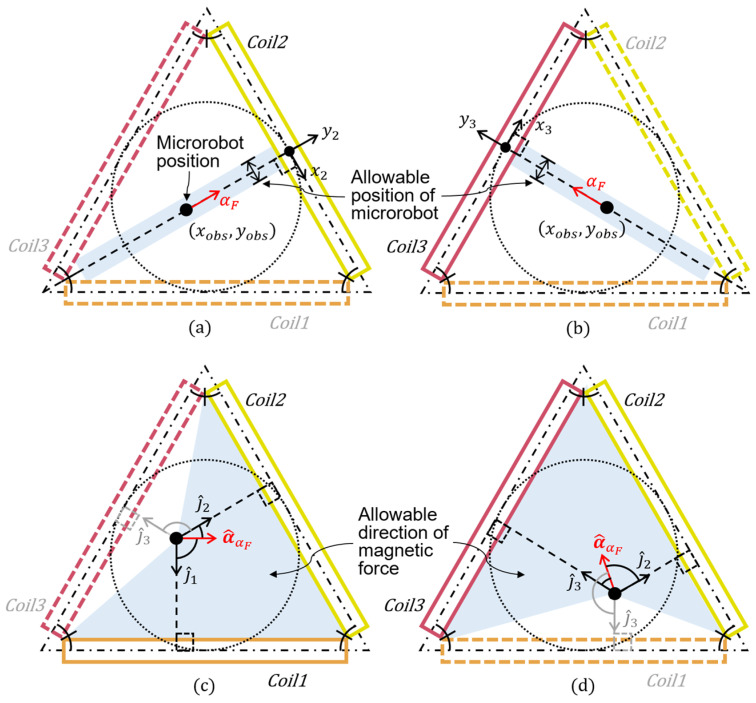
Cases showing how the microrobot can be actuated using only one of the three coils of the TEC ((**a**) *Coil 2* and (**b**) *Coil 3*) and by two coils of the TEC ((**c**) *Coil 1* and *Coil 2* and (**d**) *Coil 2* and *Coil 3*). To be actuated by only one coil of the TEC, a microrobot must be located within the allowable region.

The first condition is where only one of the three coils of the TEC is selectively used to actuate the microrobot. Near the central axis of a circular coil (a virtual magnet), unlike other positions, the magnetic field is distributed along the central axial-direction of the coil, and its magnetic gradient is also aligned in that direction. Thus, activating only one of the three coils of the TEC will cause the microrobot to be aligned with the central-axial direction of the corresponding coil, thereby actuating the microrobot along that direction. In this way, the microrobot can be moved along the central-axial direction of one of the three coils of the TEC. For example, when the microrobot is located within the allowable region of the central-axial direction of *Coil 2* (Figure 2a) or *Coil 3* (Figure 2b), it can be actuated along the respective central-axial direction by activating only the corresponding coil. With this approach, the microrobot can be easily controlled, and its movement is not affected by any coils or magnetic field other than the activated coil, resulting in improved actuation precision and efficiency. To actuate a microrobot using one of the three coils of the TEC, the operating coil can be selected as follows.

(10) $$Coil^{I}=Coil\;k\;if\;\;\alpha_{F}=\frac{4k+5}{6}\pi \;and\;\frac{y_{obs}}{x_{obs}}≅\tan\alpha_{F}.$$

Here, ${Coil}^{I}$, Coilk, $x_{obs}$, and $y_{obs}$ refer to the selected operating coil of the TEC, the *k*th coil of the TEC, and the observed coordinates in the *x*-axis and *y*-axis directions of the microrobot with respect to the global coordinate system, respectively. The tolerance margin applied to the condition in Equation (10) can be adjusted according to the required manipulation accuracy of the microrobot. Here, the magnetic force generated by the *k*th coil can be expressed as follows:

(11) $$F_{\mathrm{TEC},I}^{\mathrm{xy}}=\left[\begin{array}{c} F_{0}\cos\alpha_{F}\\ F_{0}\sin\alpha_{F} \end{array}\right]=\left[\begin{array}{c} A_{F}^{x_{k}}\left(x\right)\\ A_{F}^{y_{k}}\left(x\right) \end{array}\right]i_{k}=A_{\mathrm{Coi}l^{I}}\left(x\right)i_{Coil^{I}}.$$

Given that the actuation matrix $A_{{Coil}^{I}}$ in the equation above is determined only by the magnetic field generated by a single circular coil, the input current $i_{k}$ for the single coil required to control the actuation force of the microrobot can be analytically calculated with respect to the given x, $F_{0}$, and $\alpha_{F}$ values. However, practical applications of this case are limited, because the position and moving direction of the microrobot must be ensured to simultaneously meet the two conditions shown in (10).

The second condition is where two of the three coils of the TEC are selectively used to actuate the microrobot. This can be employed when the microrobot is located in a random position within a plane without meeting the conditions described in (10), as shown in Figure 2c,d. Previously, it was assumed that the actuation force of a microrobot could be generated by using a combination of attractions exerted by the three virtual magnets (coils) of the TEC. According to this mechanism, the 2D actuation force of a microrobot can be generated by selecting two of the three coils of the TEC and then optimizing combinations of the attractions exerted by the two coils. In this approach, based on the principle of vector summation, the smaller the deviation angle between the desired magnetic force direction for the microrobot and the central-axial direction of each coil, the higher the efficiency when generating the actuation force of the microrobot. Therefore, it is most effective to exclude the coil whose deviation angle between the desired magnetic-force direction for the microrobot and its central-axial direction is the largest, and use the other two to generate the actuation force, as follows:

(12) $$Coil^{II}=Coil\;k\;for\;k\neq \underset{l=1,2,3}{\mathrm{argmax}}\;\;\cos^{-1}\left({\hat{a}}_{\alpha_{F}}\cdot {\hat{J}}_{l}\right).$$

Here, ${Coil}^{II}$, ${\hat{a}}_{\alpha_{F}}$, and ${\hat{J}}_{l}$ refer to the two selected operating coils of the TEC, the unit vector in the direction ($\alpha_{F}$) of the magnetic force to be exerted on the microrobot, and the unit vector in the direction of the central axis of the TEC’s *l*th coil. If the *p*th and *q*th coils have been selected according to (12), the magnetic force generated by the TEC can be expressed as follows:

(13) $$F_{\mathrm{TEC},\mathrm{II}}^{\mathrm{xy}}=\left[\begin{array}{c} F_{0}\cos\alpha_{F}\\ F_{0}\sin\alpha_{F} \end{array}\right]=\left[\begin{array}{cc} A_{F}^{x_{p}}\left(x\right) & A_{F}^{x_{q}}\left(x\right)\\ A_{F}^{y_{p}}\left(x\right) & A_{F}^{y_{q}}\left(x\right) \end{array}\right]\left[\begin{array}{c} i_{p}\\ i_{q} \end{array}\right]=A_{\mathrm{Coi}l^{\mathrm{II}}}\left(x\right)I_{{Coil}^{II}}.$$

The minimum energy consumption condition can be further applied, as shown in the equation below, to numerically calculate the local minimum for the $I_{{Coil}^{II}}$ in (13):

(14) $$I_{\mathrm{Coi}l^{\mathrm{II}}}=\underset{I_{\mathrm{Coi}l^{\mathrm{II}}}\in Cons.}{\mathrm{argmin}}{\left\|I_{\mathrm{Coi}l^{\mathrm{II}}}\left(x,F_{0},\alpha_{F}\right)\right\|}^{2}.$$

Here, $I_{Coil^{II}}$ refers to ${\left[\begin{array}{cc} i_{p} & i_{q} \end{array}\right]}^{T}$, while *Cons.* refer to the constraints of $I_{Coil^{II}}$ as follows:

(15) $$Cons.=\left\{\begin{array}{c} -i_{\mathrm{allow}}\leq i_{p},i_{q}\leq i_{\mathrm{allow}}\\ F_{0}-\left\|F_{\mathrm{TEC},\mathrm{II}}^{\mathrm{xy}}\left(x,i_{p},i_{q}\right)\right\|≅0\\ \alpha_{F}-∠F_{\mathrm{TEC},\mathrm{II}}^{\mathrm{xy}}\left(x,i_{p},i_{q}\right)≅0 \end{array}\right..$$

**Figure 3:**
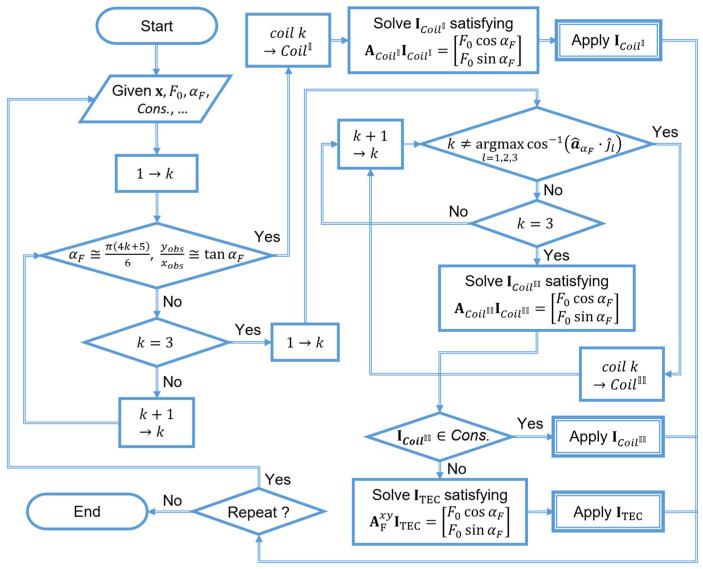
Flowchart showing how the TEC’s current is calculated and applied based on the proposed selective magnetic field generation method. Different combinations of coils may be used in each iteration.

Here, $i_{\mathrm{allow}}$ indicates the maximum allowable current for each coil of the TEC, which is determined based on the electrical specifications of the power source and the wires used in the coils of the TEC. Given that a TEC is composed of circular coils of the same kind, its overall energy consumption is assumed to be proportional to the square of the current estimated, using (14). It is worth noting that proper solutions of (13) and (14) may not exist depending on the value of $x,\;F_{0},\;\alpha_{F}$. If the input current estimated according to (13) and (14) exceeds the maximum allowable current for each coil, then this means that it may be difficult to generate sufficient actuation force on the microrobot using only two coils of the TEC. If this is the case, all three coils of the TEC could be activated using (9) to obtain another local minimum ($I_{TEC}$). This would allow the input current for each coil to fall within the maximum allowable range, thereby achieving the desired level of actuation force. Here, the constraints are implemented by applying $I_{TEC}$ to (14) and (15). Therefore, the 2D actuation force for microrobots can be generated in a precise and effective manner by selectively using the coils of a TEC while considering their current position and desired moving direction. The overall procedure for selecting the operating coil and estimating input current is schematically illustrated in Figure 3. A microrobot can be moved along desired directions by repeating the procedure shown in Figure 3, and the required actuation force can be effectively generated at each iteration.

## 3. Results and Discussion

### 3.1. Experimental Setup

**Figure 4:**
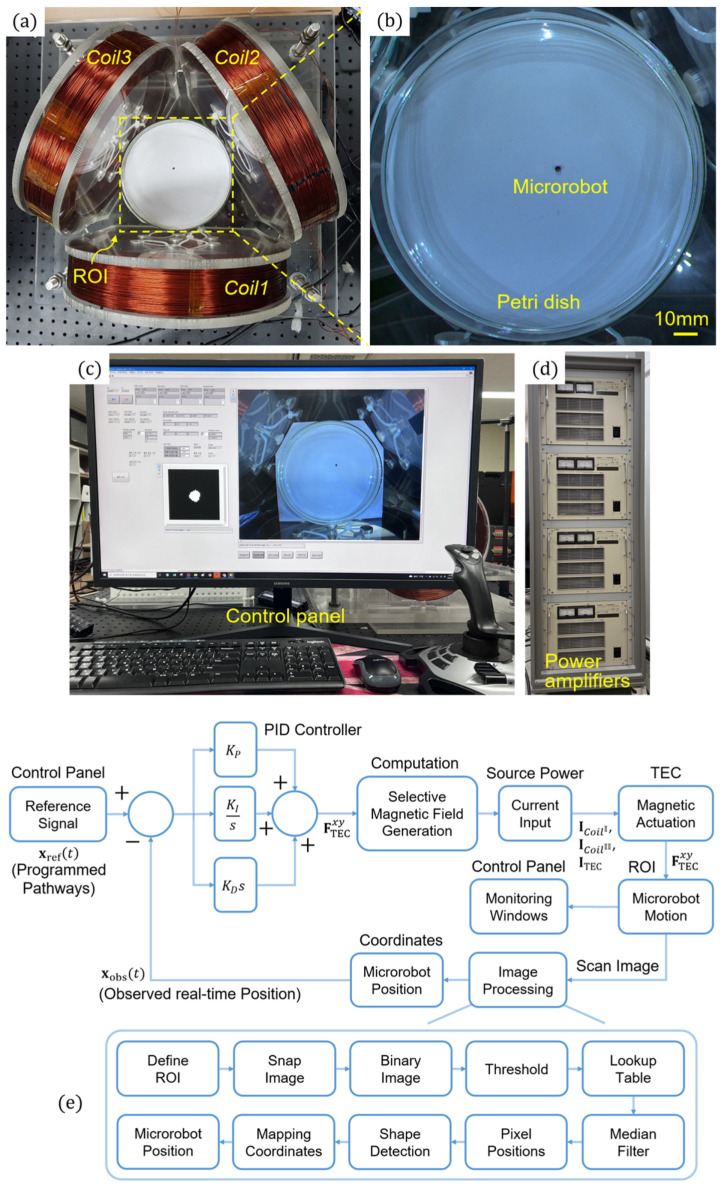
Experimental setup of the (**a**) TEC, (**b**) microrobot, (**c**) control panel, (**d**) power amplifiers, and (**e**) their integrated closed-loop control procedure. A relatively large and simple disk-type magnet was used as a microrobot to minimize the image processing time required for the real-time closed-loop control of the microrobot. The proposed magnetic field generation method shown in Figure 3 is implemented in the control procedure.

An experimental environment was established, as shown in Figure 4, to verify the validity of the TEC magnetic field generation and microrobot actuation method proposed in the present study. Figure 4 shows a prototype TEC composed of circular coils with a radius of 125 mm and 1300 turns of copper wire. Each coil of the TEC was connected to the LabVIEW hardware interface (PCle-6738, National Instruments, Austin, TX, USA) of a PC, and a power amplifier (Precision Power Amplifier 4510, NF Corporation, Yokohama, Japan) was used as the power supply source. (1)–(15) were implemented using the graphical programming platform LabVIEW 2019. To minimize the required computing time and thus improve the precision of the actuation of the microrobot, the coils used in the TEC and their input current values were calculated in advance for various actuation conditions. These predetermined values were loaded in real time whenever necessary, while considering the current position and moving direction of the microrobot.

The maximum voltage and maximum input current for the TEC were ±150 V and ±4 A, respectively. A proportional-integral-derivative (PID) controller was also employed to implement the closed-loop control of the microrobot. The gains of the proportional controller, integral controller, and differential controller were fixed at 4 $kg/s^{2}$, 3 $kg/s^{3}$, and 0.002 kg/s, respectively. In the process, a scanning camera (acA2040-120uc, Basler AG, Ahrensburg, Germany) with a maximum frame rate of 120 Hz was used to obtain real-time *xy*-plane images of the microrobot, and the resultant images were then processed using the LabVIEW Vision Assistant to obtain real-time position information. The average iteration interval of the overall system for the real-time control of a microrobot, including the acquisition and processing of microrobot images, position calculation, magnetic field application, and microrobot movement, was about 70 ms (Figure 4e).

The microrobot was prepared using a transversely magnetized N45 NdFeB magnet in the form of a disk with a diameter of 3 mm and a height of 1 mm. For the present study, the microrobot was designed to have a relatively large and simple shape, considering its large overall ROI, the scan camera’s resolution, and the processing speed of the image processing system. In future studies, the microrobots can be designed in a more compact or sophisticated manner to suit the needs of their applications. The obtained microrobot was put in a Petri dish containing silicon oil with a viscosity of 100 cP to observe its 2D movements in a fluid environment.

### 3.2. Simulated Results

The magnetic field generated by the TEC using the proposed method and the distribution of the resultant magnetic force exerted on the microrobot were first examined by simulation. For a microrobot located in the position of [x,y] within a *xy*-plane, the input currents of the TEC’s coils were set so that a constant magnetic force (0.7 mN) could be applied to the microrobot, regardless of the microrobot’s position and the number of activated coils. Detailed parameters and the results of the simulation are summarized in Table 1.

**Figure 5:**
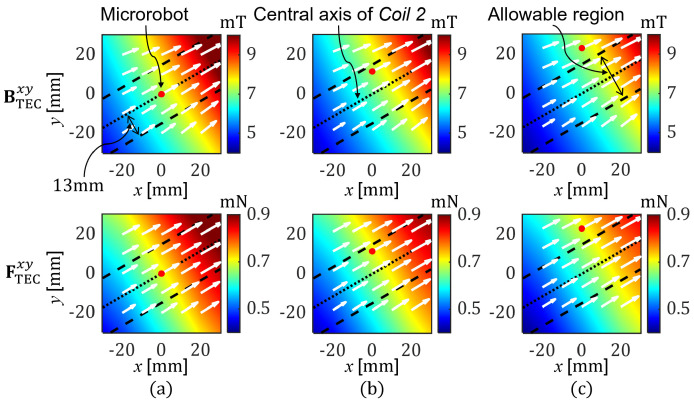
Simulated magnetic fields and the corresponding magnetic forces for the microrobot located at (**a**) [*x*,*y*] = [0,0], (**b**) [*x*,*y*] = [0,11.55], and (**c**) [*x*,*y*] = [0,23.1], generated using *Coil 2* only. The input current of *Coil 2* was calculated to keep the magnetic force at a constant magnitude of 0.7 mN regardless of the microrobot's change in position.

Figure 5 presents the distribution of the magnetic field and the corresponding magnetic force generated using a single coil (*Coil 2*) of the TEC to actuate the microrobot located on the vertical axis (*y*-axis) of the TEC. Figure 5a shows the case of $[x,y]=\left[0,0\right]$, which exactly satisfies the conditions of (10). As indicated, the microrobot can be precisely moved in the direction of 30° along the central axis of *Coil 2,* thanks to the magnetic field and the resultant magnetic force aligned in that direction. Figure 5b shows the case of $[x,y]=\left[0,11.55\right]$ (10 mm in perpendicular distance from *Coil 2*), which does not satisfy (10)’s conditions; it was found that the magnetic force was generated in the direction of 28.8°. In the present study, the tolerance angle between the desired and actual directions of the magnetic force produced using only one of the coils of the TEC was set to 1.5°. In this case, as long as the microrobot was located within a perpendicular distance of about 13 mm from the central axis of each coil, as shown in Figure 5a, the direction error of the resultant magnetic force could be kept below 1.5°, thereby enabling the microrobot to move along the axial direction of the corresponding coil. Figure 5c shows the case of $[x,y]=\left[0,23.1\right]$ (20 mm in perpendicular distance from *Coil 2*), where the microrobot is located beyond the allowable distance. For this case, it was found that the resultant magnetic force was aligned at an angle of 28.3°, i.e., beyond the allowable direction error. Given that all the coils in a TEC are symmetrically arranged, these results may be applied to *Coil 1* and *Coil 3* in the same manner. This means that the 2D actuation force can be effectively implemented and satisfy the required precision by using only one of the coils of the TEC, provided that the microrobot is located within the allowable range.

**Figure 6:**
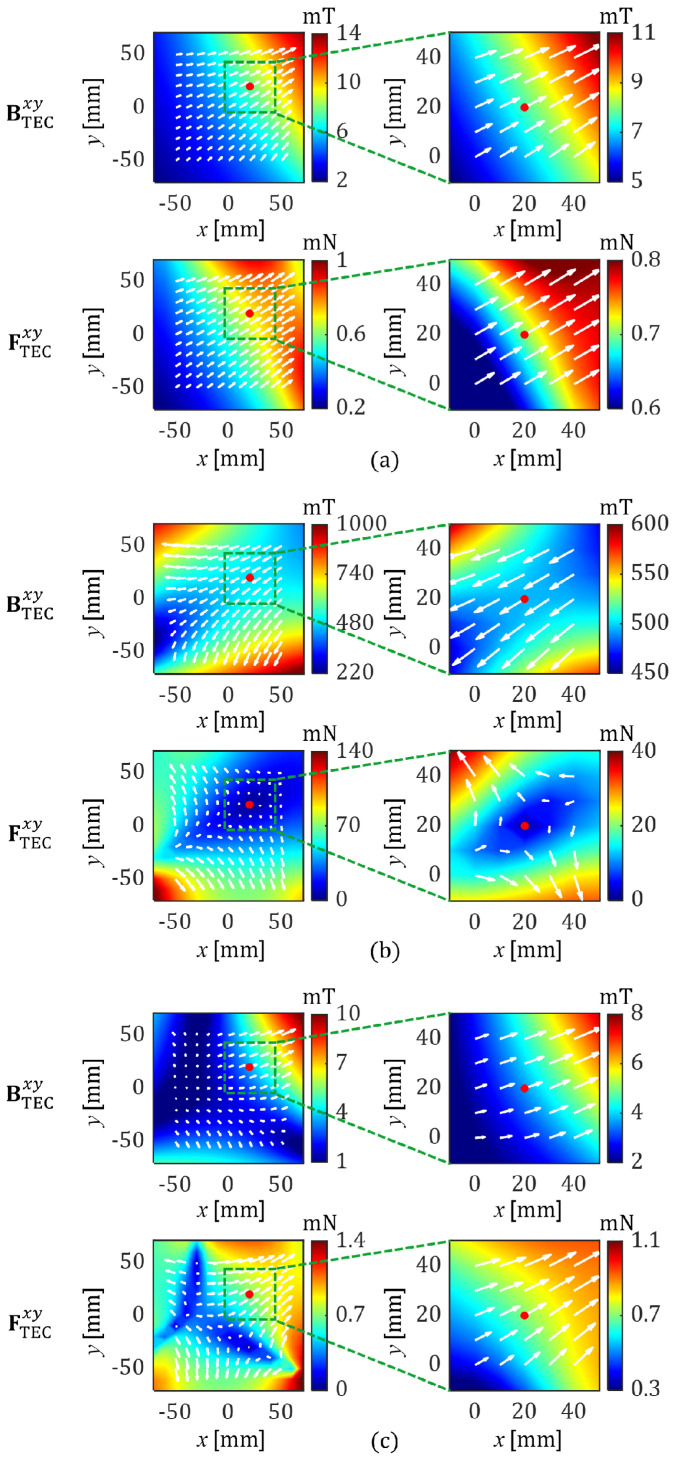
Simulated magnetic fields and the corresponding magnetic forces for the microrobot generated using (**a**) *Coil 2* and *Coil 3*, (**b**) *Coil 1* and *Coil 3*, and (**c**) *Coil 1*, *Coil 2*, and *Coil 3*. Different combinations of coils require different input currents even for the same actuation force and position of the microrobot.

Next, the case in which two or three coils of the TEC are used to generate a magnetic force was simulated. Figure 6 presents the distributions of the magnetic field and the corresponding magnetic force generated using two or three coils of the TEC to actuate the microrobot located at $[x,y]=\left[20,20\right]$ with a magnetic force of 0.7 mN in the direction of 30°. This position is not on the central axis of *Coil 2*, and thus, it is expected that using *Coil 2* alone may generate a magnetic force at an angle slightly away from 30°. However, by using a combination of two coils (*Coil 2* and *Coil 3*) selected based on the $Coil^{II}$ selection method shown in (12), it is possible to precisely apply a magnetic force at an angle of 30° to the microrobot, as shown in Figure 6a and Table 1. To show the efficacy of the selected coil combination, a different coil combination (*Coil 1* and *Coil 3*) was simulated, as shown in Figure 6b and Table 1. It showed that although the same magnetic force (0.7 mN) was applied to the microrobot, a significantly large amount of energy (79,215 $A^{2}$) must be input to the TEC if the coil combination does not comply with the proposed method.

Figure 6c shows the case in which all coils of the TEC were used (conventional method), in an attempt to apply the same magnetic force as that shown in Figure 6a. With this approach, the energy consumption was about 108% greater than when only two coils were used, as shown in Figure 6a and Table 1. These results indicate that it is more efficient to selectively use part of the coils of the TEC for the proposed method, rather than using all of them all the time, when generating a 2D actuation force for microrobots.

### 3.3. Closed-Loop Manipulation of the Microrobot

To demonstrate the validity of the proposed method of selectively using coils of the TEC for microrobot actuation, the measured actuation precision and energy consumption for various paths were compared with results obtained using all of the TECs (the conventional method). The microrobot was manipulated in a closed-loop manner to follow each given path at an average speed of 1 mm/s. Each path’s measurements were repeated five times, and the results were averaged.

**Figure 7:**
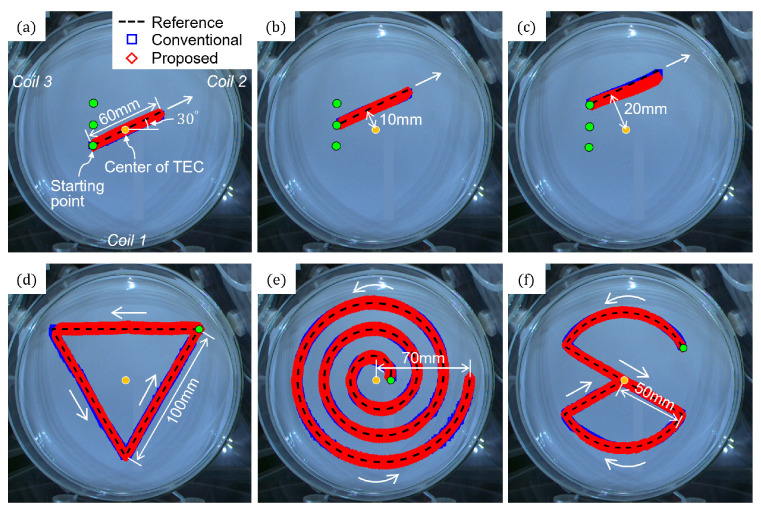
Closed-loop manipulation of the microrobot along different programmed paths using the proposed and conventional magnetic field generation methods. The figures show overlapped images of the microrobot’s marked position. In the proposed method, only one coil (*Coil 2*) of the TEC was used to manipulate the microrobot along (**a**–**c**) the straight lines, while one, two, or three coils of the TEC were selectively used to manipulate the microrobot along the paths with (**d**) inverted triangular, (**e**) spiral, and (**f**) arcs and straight-lines combined shapes (Please see the Supplementary Video S1).

**Figure 8:**
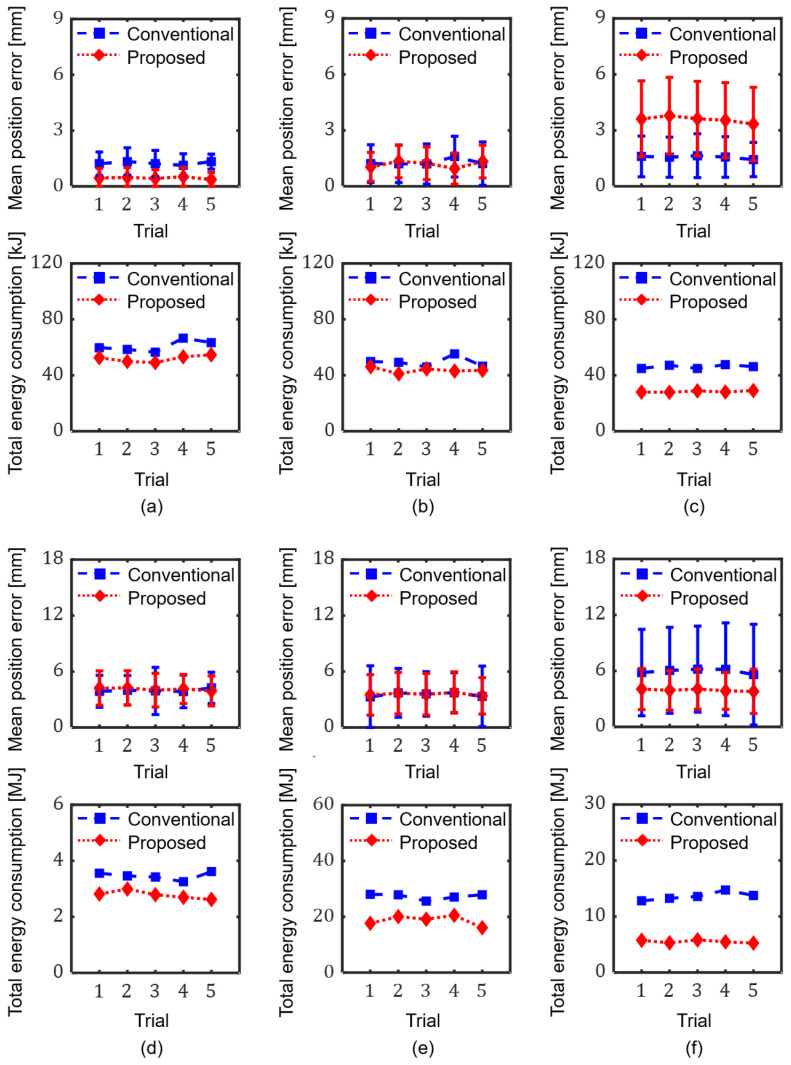
Position error and energy consumption of the conventional and proposed methods at the closed-loop test motions of the microrobot along the designated paths described in Figure 7. (**a**–**f**) present the experimental results corresponding to the paths illustrated in Figure 7a–f, respectively.

First, the cases where the proposed method used only one coil of the TEC (*Coil 2*) for three parallel straight paths were compared with those of the conventional method, as shown in Figure 7a–c. All the paths were inclined in the direction of 30°. When tested on the first straight path, which matched the central axis of *Coil 2* (Figure 5a and Figure 7a), the proposed method exhibited a much smaller average position error of 0.45 mm than the conventional method (1.25 mm), as shown in Figure 8a. When tested on the next straight path with a perpendicular distance of 10 mm from the first path (Figure 5b and Figure 7b), the proposed and conventional methods exhibited similar average errors of 1.18 mm and 1.28 mm, respectively, as shown in Figure 8b. When tested on the third straight path with a perpendicular distance of 20 mm from the first path (Figure 5c and Figure 7c), however, the proposed method showed a significantly increased average error of 3.58 mm, while the conventional method showed a slightly increased error of 1.56 mm, as shown in Figure 8c. For the microrobot outside the allowable range, as in the case shown in Figure 5c, the magnetic field generated solely by *Coil 2* cannot precisely generate the same magnetic force of as that shown in Figure 7a. This appears to be an error in the generation of the microrobot’s subsequent magnetic force, so that the microrobot’s cumulative position error gradually increases as the microrobot moves. These results show that using only one coil of the TEC should be limited to cases that satisfy the conditions shown in (10).

On the other hand, Figure 8 shows that the proposed method consumes less electric energy (20.0% on average) than the conventional method in all cases, regardless of the magnitude of the position error. When the magnetic force on the microrobot is generated using two or three coils of the TEC, more total energy is required than when the same magnetic force is generated using only one coil. This is due to the offset effect of the magnetic field between the TEC’s coils, which can be seen in Figure 5 and Figure 6, and Table 1.

Next, the cases where the proposed method selectively used coils of the TEC for microrobot actuation (Figure 3) for various paths were compared with those of the conventional method. The tolerance angle when using only one coil of the TEC was set to 1.5°. First, when tested for a path in the form of an inverted triangle, as shown in Figure 7d and Figure 8d, the average errors of the conventional and proposed methods were 3.98 mm and 4.11 mm, respectively. The closed-loop manipulation of the microrobot along this path required about 1517 iterations of input current calculation. In the proposed method, the number of one-coil, two-coil, and three-coil operations were 1, 1513, and 3, respectively, while the conventional method used all three coils at all times. Since the path in Figure 7d is composed of sub-paths, each of which is perpendicular to the central axis of each coil, it was found that one-coil operations were very rare. The average energy consumption of the conventional method was 3.46 MJ, about 24.5% larger than that of the proposed method, at 2.78 MJ.

When tested for a more complicated spiral-like path, as shown in Figure 7e and Figure 8e, the conventional and proposed methods exhibited average errors of 3.53 mm and 3.58 mm, respectively, with average standard deviations of 2.75 mm and 2.16 mm, respectively. In the proposed method, the number of one-coil, two-coil, and three-coil operations were 0, 2387, and 520, respectively. Since the tangential direction of the path in Figure 7e is almost perpendicular to the central axis of each coil, no one-coil operation was observed in this case. The average energy consumption of the conventional and proposed methods was 27.30 MJ and 18.70 MJ, respectively.

Finally, when tested for the path composed of arcs and straight lines, as shown in Figure 7f and Figure 8f, the conventional and proposed methods exhibited an average error of 5.97 mm and 3.94 mm, respectively, with average standard deviations of 4.85 mm and 2.17 mm, respectively. As illustrated in Figure 8f, the target trajectory includes frequent abrupt directional changes. This kinetic complexity induced larger positional errors compared to other trials, particularly under the conventional method utilizing all three coils. In the proposed method, the number of one-coil, two-coil, and three-coil operations were 49, 1439, and 230, respectively. The average energy consumption of the conventional method was 13.63 MJ, about 146.4% larger than that of the proposed method at 5.53 MJ, indicating the superiority of the proposed method. Although the degrees of position error and the amount of electric energy required for the robot manipulation were sometimes found to vary depending on various parameters, such as the size, ROI, and movement speed of the microrobot and the electrical specifications of the MNS, these results confirm that the 2D actuation of microrobots can be implemented in a precise and efficient manner by applying the proposed method of selectively using part of the coils of a TEC.

## 4. Conclusions

The present study proposes a system configuration, a magnetic field generation method, and a calculation procedure, which is considered to be one of the most effective magnetic robot actuation strategies so far in terms of compact structure, low energy consumption, and high manipulation accuracy. In the proposed method, part of the coils of the TEC was selectively used to generate the actuation force on the microrobot, and the results showed that the application of the proposed method led to an improvement in actuation accuracy and manipulation efficiency. The TEC can be built at relatively low cost using three fixed circular coils, and its symmetrical arrangement facilitates a microrobot’s intuitive and straightforward manipulation. System optimization for micro-scale actuation and subsequent experimental verification remain crucial areas for future research. Furthermore, the proposed system can be advanced to support enhanced functionality and higher degrees of freedom manipulation. Ultimately, the major findings of this study are expected to contribute to the development of future technologies capable of effectively actuating microrobots using a structurally simple and magnetically efficient MNS.

## Figures and Tables

**Table 1:** Detailed simulation results of the magnetic fields and magnetic forces applied to the microrobots in Figure 5 and Figure 6.

Figures (Selected Coils)	Position ([*x*, *y*]) [mm]	$\left\|B_{TEC}^{xy}\right\|$ [mT]	$∠B_{TEC}^{xy}$ [deg.]	$\left\|F_{TEC}^{xy}\right\|$ [mN]	$∠F_{TEC}^{xy}$ [deg.]	$I_{TEC}$ [A]	${\left\|I_{TEC}\right\|}^{2}$ $[A^{2}$]
Figure 5a (*Coil 2*)	$\left[0,\;0\right]$	7.2	30.0	0.7	30.0	${\left[\begin{array}{ccc} 0 & 3.01 & 0 \end{array}\right]}^{T}$	9.06
Figure 5b (*Coil 2*)	$\left[0,\;11.55\right]$	7.3	26.9	0.7	28.8	${\left[\begin{array}{ccc} 0 & 2.84 & 0 \end{array}\right]}^{T}$	8.07
Figure 5c (*Coil 2*)	$\left[0,\;23.1\right]$	7.3	23.8	0.7	28.3	${\left[\begin{array}{ccc} 0 & 2.67 & 0 \end{array}\right]}^{T}$	7.14
Figure 6a (*Coil 2*, *Coil 3*)	$\left[20,\;20\right]$	7.7	27.6	0.7	30.0	${\left[\begin{array}{ccc} 0 & 2.37 & -0.02 \end{array}\right]}^{T}$	5.62
Figure 6b (*Coil 1*, *Coil 3*)	$\left[20,\;20\right]$	495.3	211.2	0.7	30.0	${\left[\begin{array}{ccc} 211.80 & 0 & 185.40 \end{array}\right]}^{T}$	79,215
Figure 6c (*Coil 1*, *Coil 2*, *Coil 3*)	$\left[20,\;20\right]$	3.7	20.0	0.7	30.0	${\left[\begin{array}{ccc} 1.88 & 2.43 & 1.50 \end{array}\right]}^{T}$	11.69

## Data Availability

The data that support the findings of this study are available from the corresponding author upon reasonable request.

## Supplementary Materials

The following supporting information can be downloaded at https://www.mdpi.com/article/10.3390/mi17030337/s1, Video S1: Closed-loop manipulation of the microrobot (Figure 7).
